# Supplementary material for: Combined Transcriptomic and Protein Array Cytokine Profiling of Human Stem Cells from Dental Apical Papilla Modulated by Oral Bacteria
Source: Int J Mol Sci. 2022 May 3;23(9):5098. doi: 10.3390/ijms23095098 (PMC9103834; doi:10.3390/ijms23095098)
Supplement: Supplementary file 1 [file ijms-23-05098-s001.zip › ijms-1695399-supplementary.pdf]

Supplementary Materials

1.1 SCAPs exposed to viable F. nucleatum and E. faecalis as well as F. nucleatum supernatants at a concentration of MOI 100 expressed visible effects on SCAPs proliferation.

Concentration sufficient to inhibit cell proliferation by 50% (IC50) after 24 hours SCAP-bacteria co-culture was calculated using the xCELLigence Real Time Cell Analyser (RTCA, Roche Diagnostics GmbH, Mannheim, Germany). Taken together, only SCAPs exposed to viable *F. nucleatum* and *E. faecalis* as well as *F. nucleatum* supernatants at a concentration of MOI 100 expressed visible effects ($p<0.05$) and were chosen for this study (Supplements S1).

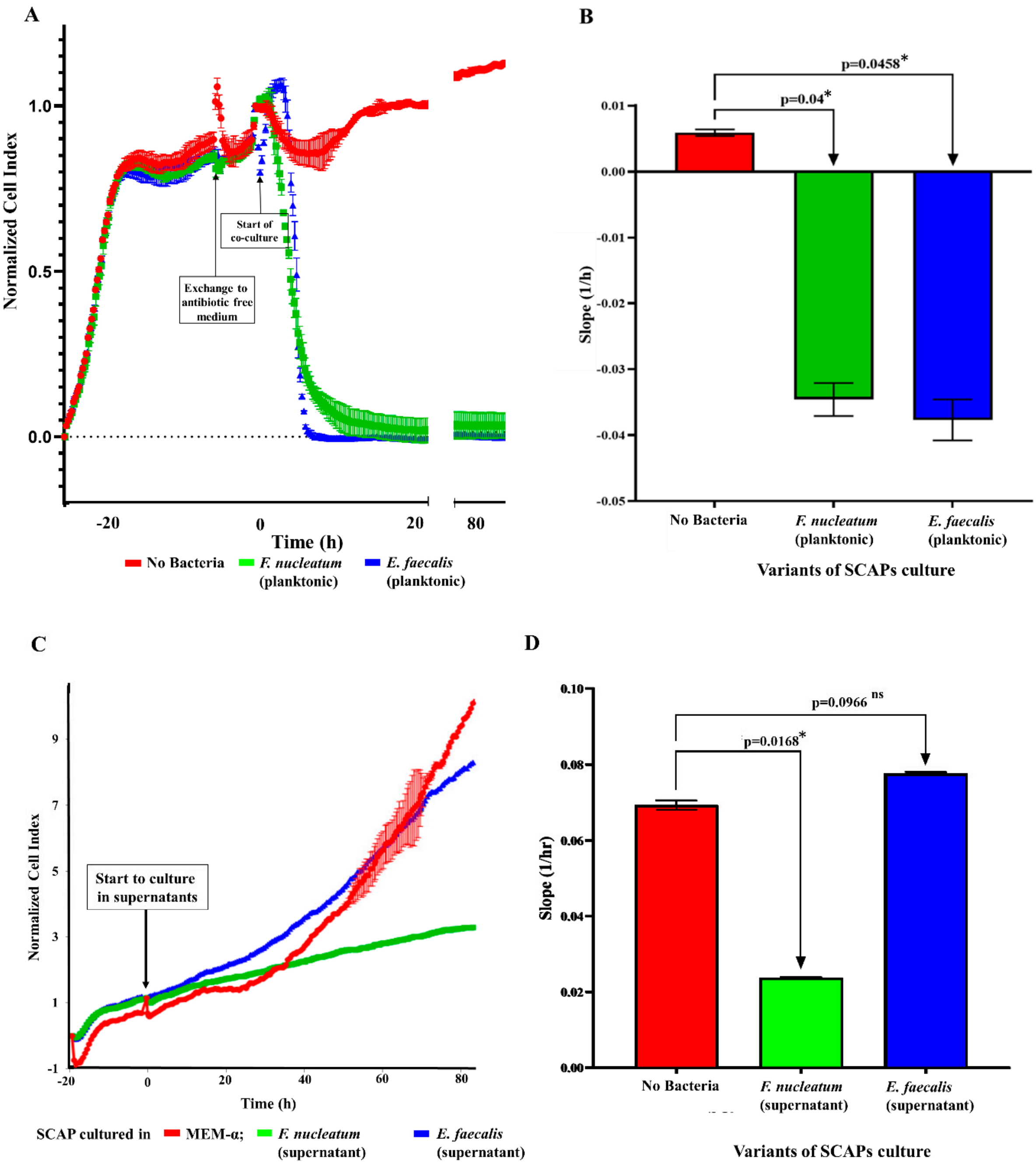

Figure S1. Monitoring SCAP modulated by oral bacteria in Real Time. Only SCAPs exposed to viable *F. nucleatum* and *E. faecalis* as well as *F. nucleatum* supernatants at a concentration of MOI 100 expressed visible effects on proliferation. SCAP proliferation curves in different variants of co-culture with bacteria at planktonic stage (A) or cultured in supernatants of *F. nucleatum* or *E. faecalis* (C); (B and D) – the slopes of corresponding proliferation curves calculated by the RTCA instrument software according to the formula: slope multiplied by time with addition of intercept, the calculation was set up on the time of co-culture / treatment. Statistical analysis of slopes was performed using the GraphPad Prism 8.4.3 (GraphPad Software Inc. San Diego, USA) software package using the Brown-Forsythe and Welch analysis of variance (ANOVA) tests and multiple comparison tests, significance set at $p < 0.05$.

Table S1. List of the capture antibodies spotted on nitrocellulose membrane against the designated cytokines, chemokines or acute phase proteins (RnD systems, ARY022B).

Coordinate on the membrane	Analyte/Control	Alternate Nomenclature	Gene ID
A1, A2	Reference Spots	RS	N/A
A3, A4	**Adiponectin**	Acrp30	9370
A5, A6	**Apolipoprotein A-I**	ApoA1	335
A7, A8	**Angiogenin**	______	283
A9, A10	**Angiopoietin-1**	Ang-1, ANGPT1	284
A11, A12	**Angiopoietin-2**	Ang-2, ANGPT2	285
A13, A14	**BAFF**	BLyS, TNFSF13B	10673
A15, A16	**BDNF**	Brain-derived Neurotrophic Factor	627
A17, A18	**Complement Component C5/C5a**	C5/C5a	727
A19, A20	**CD14**	______	929
A21, A22	**CD30**	TNFRSF8	943
A23, A24	Reference Spots	RS	N/A
B3, B4	**CD40 ligand**	CD40L, TNFSF5, CD154, TRAP	959
B5, B6	**Chitinase 3-like 1**	CHI3L1, YKL-40	1116
B7, B8	**Complement Factor D**	Adipsin, CFD	1675
B9, B10	**C-Reactive Protein**	CRP	1401
B11, B12	**Cripto-1**	Teratocarcinoma-derived Growth Factor	6997
B13, B14	**Cystatin C**	CST3, ARMD11	1471
B15, B16	**Dkk-1**	Dickkopf-1	22943
B17, B18	**DPPIV**	CD26, DPP4, Dipeptidyl-peptidase IV	1803
B19, B20	**EGF**	Epidermal Growth Factor	1950
B21, B22	**Emmprin**	CD147, Basigin	682
C3, C4	**ENA-78**	CXCL5	6374
C5, C6	**Endoglin**	CD105, ENG	2022
C7, C8	**Fas Ligand**	TNFSF6, CD178, CD95L	356
C9, C10	**FGF basic**	FGF-2	2247
C11, C12	**FGF-7**	KGF	2252
C13, C14	**FGF-19**	______	9965

Coordinate on the membrane	Analyte/Control	Alternate Nomenclature	Gene ID
C15, C16	**Flt-3 Ligand**	FLT3LG	2323
C17, C18	**G-CSF**	CSF3	1440
C19, C20	**GDF-15**	MIC-1	9518
C21, C22	**GM-CSF**	CSF2	1437
D1, D2	**GROα**	CXCL1, MSGA-α	2919
D3, D4	**Growth Hormone**	GH, Somatotropin	2688
D5, D6	**HGF**	Scatter Factor, SF	3082
D7, D8	**ICAM-1**	CD54	3383
D9, D10	**IFN-γ**	IFNG	3458
D11, D12	**IGFBP-2**	______	3485
D13, D14	**IGFBP-3**	______	3486
D15, D16	**IL-1α**	IL-1F1	3552
D17, D18	**IL-1β**	IL-1F2	3553
D19, D20	**IL-1ra**	IL-1F3	3557
D21, D22	**IL-2**	______	3558
D23, D24	**IL-3**	______	3562
E1, E2	**IL-4**	______	3565
E3, E4	**IL-5**	______	3567
E5, E6	**IL-6**	______	3569
E7, E8	**IL-8**	CXCL8	3576
E9, E10	**IL-10**	______	3586
E11, E12	**IL-11**	______	3589
E13, E14	**IL-12 p70**	______	3593
E15, E16	**IL-13**	______	3596
E17, E18	**IL-15**	______	3600
E19, E20	**IL-16**	______	3603
E21, E22	**IL-17A**	IL-17, CTLA8	3605
E23, E24	**IL-18 Bpa**	______	10068
F1, F2	**IL-19**	______	29949
F3, F4	**IL-22**	IL-TIF	50616
F5, F6	**IL-23**	IL-23A, SGRF	51561
F7, F8	**IL-24**	C49A, FISP, MDA-7, MOB-5, ST16	11009
F9, F10	**IL-27**	______	246778
F11, F12	**IL-31**	______	386653
F13, F14	**IL-32**	______	9235
F15, F16	**IL-33**	C9orf26, DVS27, NF-HEV	90865
F17, F18	**IL-34**	C16orf77	146433
F19, F20	**IP-10**	CXCL10	3627

Coordinate on the membrane	Analyte/Control	Alternate Nomenclature	Gene ID
F21, F22	**I-TAC**	CXCL11, SCYB9B	6373
F23, F24	**Kallikrein 3**	PSA, KLK3	354
G1, G2	**Leptin**	OB	3952
G3, G4	**LIF**	______	3976
G5, G6	**Lipocalin-2**	NGAL, LCN2, Siderocalin	3934
G7, G8	**MCP-1**	CCL2, MCAF	6347
G9, G10	**MCP-3**	CCL7, MARC	6354
G11, G12	**M-CSF**	CSF1	1435
G13, G14	**MIF**	______	4282
G15, G16	**MIG**	CXCL9	4283
G17, G18	**MIP-1α/MIP-1β**	CCL3/CCL4	6348/6351
G19, G20	**MIP-3α**	CCL20, Exodus-1, LARC	6364
G21, G22	**MIP-3β**	CCL19, ELC	6363
G23, G24	**MMP-9**	CLG4B, Gelatinase B	4318
H1, H2	**Myeloperoxidase**	MPO, Lactoperoxidase	4353
H3, H4	**Osteopontin**	OPN	6696
H5, H6	**PDGF-AA**	______	5154
H7, H8	**PDGF-AB/BB**	______	5154/5155
H9, H10	**Pentraxin 3**	PTX3, TSG-14	5806
H11, H12	**PF4**	CXCL4	5196
H13, H14	**RAGE**	______	177
H15, H16	**RANTES**	CCL5	6352
H17, H18	**RBP-4**	______	5950
H19, H20	**Relaxin-2**	RLN2, RLXH2	6019
H21, H22	**Resistin**	ADSF, FIZZ3, RETN	56729
H23, H24	**SDF-1α**	CXCL12, PBSF	6387
I1, I2	**Serpin E1**	PAI-I, PAI-1, Nexin	5054
I3, I4	**SHBG**	ABP	6462
I5, I6	**ST2**	IL-1 R4, IL1RL1, ST2L	9173
I7, I8	**TARC**	CCL17	6361
I9, I10	**TFF3**	ITF, TFI	7033
I11, I12	**TfR**	CD71, TFR1, TFRC, TRFR	7037
I13, I14	**TGF-α**	TGFA	7039
I15, I16	**Thrombospondin-1**	THBS1, TSP-1	7057
I17, I18	**TNF-α**	TNFSF1A	7124
I19, I20	**uPAR**	PLAUR	5329
I21, I22	**VEGF**	BEGFA	7422
J1, J2	Reference Spots	RS	N/A

Coordinate on the membrane	Analyte/Control	Alternate Nomenclature	Gene ID
J5, J6	**Vitamin D BP**	VDB, DBP, VDBP	2638
J7, J8	**CD31**	PECAM-1	5175
J9, J10	**TIM-3**	HAVCR2	84868
J11, J12	**VCAM-1**	CD106	7412
J23, J24	Negative Controls	Control (-)	N/A
